# Assessment of heat transfer and the consequences of iron oxide (Fe_3_O_4_) nanoparticles on flow of blood in an abdominal aortic aneurysm

**DOI:** 10.1016/j.heliyon.2023.e17660

**Published:** 2023-06-28

**Authors:** Azad Hussain, Muhammad Naveel Riaz Dar, Nashmi H. Alrasheed, Khalil Hajlaoui, Mohamed Bechir Ben Hamida

**Affiliations:** a Department of Mathematics, University of Gujrat, Gujrat 50700, Pakistan; b College of Engineering, Department of Mechanical Engineering, Imam Mohammad Ibn Saud Islamic University (IMSIU), Riyadh, Saudi Arabia; c Research Laboratory of Ionized Backgrounds and Reagents Studies (EMIR), Preparatory Institute for Engineering Studies of Monastir (IPEIM), University of Monastir, Monastir City, Tunisia; d Higher School of Sciences and Technology of Hammam Sousse (ESSTHS), University of Sousse, Tunisia

**Keywords:** Incompressible, Unsteady, Laminar, Newtonian fluid, Nanoparticles (Fe_3_O_4_), CFD, Aneurysm

## Abstract

The present study is established on a simulation using CFD analysis in COMSOL. Blood acted as the base fluid with this simulation. The taken flow is been modeled as incompressible, unsteady, laminar and Newtonian fluid, which is appropriate at high rates of shear. The characteristic of flow of blood is been studied in order to determine pressure, velocity and temperature impact caused by an abdominal aortic aneurysm (AAA). This work employs nanoparticles of the Iron Oxide (Fe_3_O_4_) type. The CFD technique is utilized to evaluate the equations of mass, momentum, and energy. The COMSOL software is utilized to generate a normal element sized mesh. The findings of this study demonstrate that velocity alters through aneurysmal part of the aorta, that velocity is higher in a diseased segment, and that velocity increases before and after the aneurysmal region. For the heat transfer feature, the reference temperature and general inward heat flux is taken as 293.15K and 800W/m^2^. The nanoparticles altered blood's physical properties, including conductivity, dynamic viscosity, specific heat, and density. The inclusion of Iron Oxide (Fe_3_O_4_) nanoparticles managed to prevent overheating because taken nanoparticles have significant thermal conductivity. These findings will be extremely beneficial in the treatment of abdominal aortic aneurysm.

## Nomenclature

p: Pressure of Fluid (kgm^−1^s^−2^)
r: Radial direction (m)
t: Time (sec)
$\rho_{nf}$: Density of nanofluids (kg/m^3^)
u: Radial velocity component (m/s)
v: Tangential velocity component (ms^−1^)
μ: Fluid Reference viscosity (kg $m^{-1}s^{-1})$
X: Axial direction
ρ: Fluid Density
$U_{0}$: Normal inflow velocity (m $s^{-1})$
θ: Tangential direction (rad)
w: Axial velocity (m $s^{-1})$

## 1 Introduction

An aneurysm is a condition in which a blood vessel's diameter has risen by more than 50% in comparison to its anticipated normal diameter [1]. It is caused by an uneven growth or weakening blood artery wall that is bubble or inflated shaped. The human body frequently contains Aortic, Cerebral, and Peripheral aneurysms. Nearly 75% of people with aneurysms often away before getting to the hospital, and the cause and nature of the condition are still hotly contested issues. An aneurysm is inspected for steady state flow including a Reynold number ranging from 500 to 2600 [2]. A mathematical estimation of blood flow structures and physiologic stresses was investigated in a two-aneurysm abdominal aortic aneurysm [3]. The wall shear force for such an aortic dissection aneurysm is estimated, and it is demonstrated that the techniques are very useful for treatment methodology in vascular complications [4]. The impact of cyclic exertion on AAA (abdominal aortic aneurysm) and cardiovascular endothelial cells has been analyzed [5], and it has been found that wall calcification causes a significant change in AAA. The hemodynamic phenomenon of blood supply is influenced by the different shapes of the aneurysmal artery, according to an assessment of blood flow via aneurysm models [6]. Computational analysis on the rupture of the intracranial aneurysms of blood flow has been accomplished [7], and it signifies that the elevation of the incoming blood is greatly effective inside the volatile segment on the aortic dissection artery wall. In the diagnosis, risk assessment, treatment planning, and follow-up of AAA, blood flow is crucial. Healthcare professionals can better understand the condition and devise the best treatment plans to lower the risk of rupture and enhance patient outcomes by analyzing blood flow patterns inside the aneurysm.

**Table:** 

Causses	Signs	Health Issue
Artery hardening, High blood pressure, Diseases of the blood vessels, Trauma, aortic infection.	Constant and deep pain in the side of the belly or the belly area, Back pain, A pulse close to the bellybutton.	Aortic rupture, blood clots, blockage of blood flow to the toes, legs, abdominal organs or kidneys,

A smaller number of nanoparticles (NPs), when spread and dissolved into the conventional fluid might provide some significant improvements in the thermal characteristics of the fluid under consideration. The thermal characteristics of nanofluid are superior to those of base fluid particles. Nanotechnology-based delivery of drugs has several benefits and offers an approach for addressing issues related to conventional systems of drug delivery. Choi et al. [8] were the very first to portray this novel category of heat transfer fluid-based nanotechnology. The analysis of metallic type nanoparticles on flow of blood through curved tapered arteries analyzed by Nadeem et al. [9]. In a symmetric channel, Akbar et al. [10] examined the peristaltic incompressible flow of viscous fluid comprising nanoparticles. Ellahi et al. [11] examined the impact of nanoparticles on flow of blood by composite stenotic arteries along with permeable and slip walls. T. Hayat et al. [12] investigated heat transfer enhancement by incorporating both copper and silver nanomaterials into the working fluids water. S. Ijaz et al. [13] probed the flow of blood across numerous stenosis using single-walled carbon nanotubes with variable viscosity. In [14,15], the three-dimensional flow patterns with various effects and the addition of nanoparticles are investigated. Faqir shah et al. [16] investigated the influence of entropy for hybrid nanofluid flow towards a curvy surface. Khalid Abdulkhaliq et al. [17] examined the enhancement of heat transfer for slip flow of single and multi-walled carbon nanotubes. Iftikhar Ahmad et al. [18] explored the simulation of casson nanofluid configuration by unsteady stretched surface. P. Karmakar and S. Das et al. briefly discussed the blood flow through arteries under the effect of different nano particles in [19,20,21,22].

Nanomaterials have a great scientific importance because they support as a bridge among bulk materials and molecular or atomic structural features, and the material characteristics may change as they approach the nanoscale. The potential uses of iron oxide nanoparticles (IONPs) in biomedical imaging, drug delivery, and cancer treatment have been thoroughly investigated. The way IONPs interact with biological systems, particularly blood flow, is an important factor. For this application, iron oxide nanoparticles (Fe_3_O_4_) with more greater saturation magnetization points are ideal. Badfar et al. [23] used Iron Oxide Fe_3_O_4_ magnetic nanoparticles in a similar magnetic field to visualise drug targeting in blood vessels with stenosis. According to Chandrashekhar et al. [24], magnetic fluid hyperthermia is used to cure several blood diseases such as restenosis, which corresponds to a recurring stenosis, in order to eradicate lesion, ablate nerves, and relieve pain by increasing flow of blood in that region. Some authors conducted experiments to investigate heat transfer in a pipe with both a friction coefficient of 5 and a large Prandtl of laminar fluid flow [25]. Venkatadri demonstrated that the influence of magnetic wire locations inside a square enclosure is significant towards the impact on the fluid [26]. As an outcome, nanosized solid particles are frequently used in studies and research projects. Experiment results show that nanoparticles enhance fluid conductivity because of their high thermal conductivity and scattering with in base fluid, that is one of the basic components of heat transfer [27]. Sami Ullah Khan et al. [28,29,30] researched the water base Casson nanofluid flow through with inclined magnetic effect, nonuniform heat sink/source, thermal conductivity along with slip effect and heat generational effect.

In recent years, the CFD technique has proven useful for problems involving physiological flows. The primary goal of CFD is to numerically approximate fluid movement and heat transportation using computational process. J. Liu et al. [31] used COMSOL to investigate numerical solutions to problems governing equations. A. Hussain et al. [32] investigated the heat transportation implications of unsteady and laminar flowing fluid by different elliptic cylinders, determining the maximal and minimal values for temperature, velocity and pressure.

The phenomenon of flow of blood by an aneurysmal area is investigated in this report. We supposed blood to be a Newtonian fluid while it expresses Newtonian characteristics in sizable cavities, arteries, and veins. COMSOL Multiphysics (software) used to solve the conservation of mass, momentum, and energy, and the outcomes are then estimated for something like the velocity, temperature, and pressure of the blood flow within the infected area using CFD. This paper highlights a computational exploration of the addition of nanoparticles of Iron oxide (Fe_3_O_4_) into blood that flows through such an aneurysmal region to explore how NPs could indeed assist in improvement of blood supply.

## 2 Geometry and methods

**Fig. 1:**
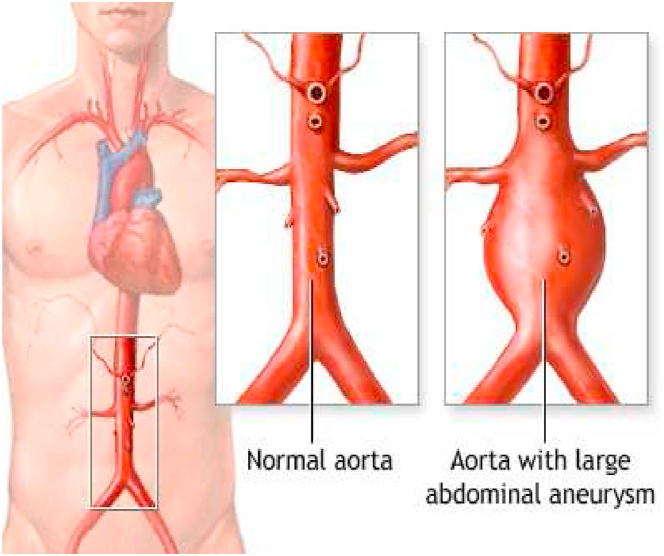
View of abdominal aortic aneurysm.

**Fig. 2:**
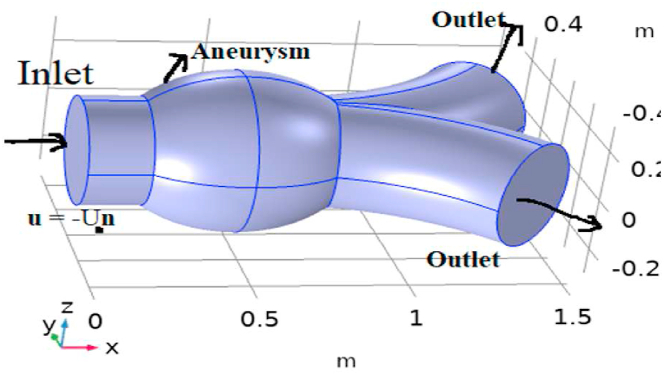
Geometric model for abdominal aortic aneurysm.

In this article, blood is taken to be unsteady and incompressible Newtonian fluid passing through AAA. The focused enthusiasm for assuming the current model is shown in Fig. 1. A geometrical sketch of blood supply problem in abdominal aortic aneurysm (AAA) is presented in Fig. 2, and the system of coordinate is chosen in the way, in which the blood is flowing in the direction of z-axis and r is taken as orthogonal to the direction of flow.

The radius and length of 2-dimensional artery is taken as 0.2 m and 1.5 m, respectively. The length is taken as from 0 to 1.5 m along the z-axis. The heat transfer and laminar blood flow physics is incorporated to COMSOL 5.4 utilizing the feature “add physics”. The velocity is 0 ms^−1^ at the boundary wall, and the exit and entrance locations are chosen. In the physics of laminar flow, the reference temperature is taken as 293.15K. For the purpose of heat flow, we selected general convective heat flux is 800W/m^2^. Blood's material properties, i.e., physical characteristics, are been appended. The time - dependent simulation model equations, namely mass, momentum, and heat transfer equations, are introduced.

Continuity equation

(1) $$\frac{\partial u}{\partial r}+\frac{u}{r}+\frac{\partial w}{\partial x}=0\text{,}$$

The equation of continuity that outlined blood mass transportation in COMSOL is written as:

(2) ∇.(u)=0,

Here u and w are indeed the nanofluid velocities in the r and z paths, respectively in equations (1), (2).

Momentum equation

(3) $$\rho_{nf}\left(\frac{\partial u}{\partial t}+u\frac{\partial u}{\partial r}+w\frac{\partial u}{\partial x}\right)=-\frac{\partial p}{\partial r}+\mu_{nf}\left(\frac{1}{r}\frac{\partial u}{\partial r}+\frac{\partial^{2}u}{{\partial r}^{2}}+\frac{\partial^{2}u}{{\partial x}^{2}}-\frac{u}{r^{2}}\right)\text{,}$$

(4) $$\rho_{nf}\left(\frac{\partial w}{\partial t}+u\frac{\partial w}{\partial r}+w\frac{\partial w}{\partial x}\right)=-\frac{\partial p}{\partial z}+\mu_{nf}\left(\frac{1}{r}\frac{\partial w}{\partial r}+\frac{\partial^{2}w}{{\partial r}^{2}}+\frac{\partial^{2}w}{{\partial x}^{2}}\right)\text{.}$$

The equation of momentum that explain blood motion in COMSOL is written as [33]:

(5) $$\rho \frac{\partial u}{\partial t}+\rho \left(u.\nabla \right)u=\mathrm{div}\left[-PI]+\mathrm{div}[S\right]+F\text{,}$$

where $S={\mu (grad\left(u\right)+\left(grad\left(u\right)\right)}^{T})$.

Here $\mu_{nf}$, *P* and $\rho_{nf}$ are the viscosity, pressure and density of the nano-fluid, respectively.

Equation of energy

(6) $${\left(\rho C_{p}\right)}_{nf}\left(\frac{\partial T}{\partial t}+u\frac{\partial w}{\partial r}+w\frac{\partial w}{\partial x}\right)=k_{nf}\left(\frac{1}{r}\frac{\partial T}{\partial r}+\frac{\partial^{2}T}{{\partial r}^{2}}+\frac{\partial^{2}T}{{\partial x}^{2}}\right)$$

The produced energy equation in COMSOL is

(7) $$\rho C_{p}\left(\frac{\partial T}{\partial t}+u.\nabla T\right)+\nabla .q=Q_{p}+Q_{vd}+Q\text{.}$$

The physical characteristics of nanofluid are written as in equ. (8) [34]:

$$\rho_{nf}=\rho_{f}\left(\left(1-\varphi \right)+\varphi \frac{\rho_{s}}{\rho_{f}}\right),\mu_{nf}=\frac{\mu_{f}}{\left(1-\varphi \right)},\frac{K_{nf}}{K_{f}}=\frac{\left(K_{s}+2K_{f}\right)-2\varphi \left(K_{f}-K_{s}\right)}{\left(K_{s}+2K_{f}\right)+\varphi \left(K_{f}-K_{s}\right)}$$

and

(8) $${\left(\rho C_{p}\right)}_{nf}={\left(\rho C_{p}\right)}_{f}\left(\left(1-\varphi \right)+\varphi \frac{{\left(\rho C_{p}\right)}_{{Fe}_{3}O_{4}}}{{\left(\rho C_{p}\right)}_{blood}}\right)\text{.}$$

Here $q=-k\nabla T,Q_{p}=\alpha_{p}T\left(\frac{\partial p}{\partial t}+u\nabla p\right),Q_{vd}=\tau .\nabla u,Q=0,\alpha_{p}=-\frac{1}{p}\frac{\partial p}{\partial t},\tau =-PI+S$, Q is the source of heat coefficient, $Q_{vd}$ is the viscous dissipation which is also zero in present case, ${\left(C_{p}\right)}_{nf}$ and ${\left(C_{p}\right)}_{f}$ represent the heat capacitance of nanofluid and base fluid, ∇T is change in temperature or temperature gradient, φ is the concentration of fluids, $k_{nf}$ and $k_{nf}$ are the value of specific heat of nano and base fluid, $\mu_{f}$ and $\mu_{nf}$ are the viscosities of base and nano fluids, and $\rho_{f}$ and $\rho_{nf}$ are the densities of base and nano fluids. We considered blood as base fluid and Iron oxide (Fe_3_O_4_) as nano fluid. The associated initial conditions are as follows:

(9) $$u=w=P=0,T=T_{0}$$

The boundary conditions are:

(10) $$u=0,w=u_{w}=\frac{{zu}_{0}}{l},T=T_{w}\;at\;r=a\;and\;w\to 0,T\to T_{\infty }\;as\;r\to \infty$$

Here w is the velocity along the flow axis which is known as stretching velocity, l is reference length and $u_{0}>0$ value of stretching rate in equation (10).

Transformation of Equations

The velocity components in the form of stream function and similarity transformations are defined as [35].

$$u=-\frac{1}{r}\frac{\partial \psi }{\partial x},w=\frac{1}{r}\frac{\partial \psi }{\partial r},\psi =a\sqrt{u_{w}\upsilon x}f\left(\eta \right),\eta =\frac{r^{2}-a^{2}}{2a}\sqrt{\frac{u_{w}}{\nu l}},u=-\frac{1}{r}\frac{\partial \psi }{\partial x}=-\sqrt{\frac{u_{0}}{\nu l}}\frac{af\left(\eta \right)}{r}\text{,}$$

(11) $$w=\frac{1}{r}\frac{\partial \psi }{\partial x}=\frac{u_{0}\nu }{l}\;f\left(\eta \right),P\left(\xi ,\eta \right)=\frac{u_{0}}{l}\mu \left(P_{1}+\xi^{2}P_{2}\right),\theta \left(\xi ,\eta \right)=\frac{T_{\infty }-T}{T_{\infty }-T_{w}}=\theta_{1}+\xi^{2}\theta_{2},\xi =\frac{x}{l}\;\text{.}$$

Clearly, the continuity equation satisfied. By using equation (11) in equations (3), (4), (5), (6), (7), we obtain the following non-linear ODE's (12–16)

(12) $${\left(1+2\eta \gamma \right)}^{2}\frac{\partial P_{1}}{\partial \eta }+\frac{1}{C_{1}C_{2}}{\left(1+2\eta \gamma \right)}^{2}f^{\prime \prime }+\left(1+2\eta \gamma \right)ff^{\prime }-\gamma f^{2}=0\text{,}$$

(13) $$\frac{1}{C_{1}C_{2}}\left[\left(1+2\eta \gamma \right)f^{\prime \prime \prime }+2\gamma f^{\prime \prime }\right]+ff^{\prime \prime }-\frac{2}{u_{0}}P_{2}=0\text{,}$$

(14) $${\left(1+2\eta \gamma \right)}^{2}\frac{\partial P_{2}}{\partial \eta }=0\text{,}$$

(15) $$\frac{1}{C_{3}\;\mathrm{Pr}}\left[\left(1+2\eta \gamma \right)l^{2}\theta_{1}^{\prime \prime }+2l^{2}\gamma \theta_{1}^{\prime }+a^{2}\gamma^{2}\theta_{2}\right]+\theta_{1}^{\prime }f=0\text{,}$$

(16) $$\frac{1}{C_{3}\;\mathrm{Pr}}\left[\left(1+2\eta \gamma \right)al^{2}{\gamma \theta }_{2}^{\prime \prime }+2l^{2}\gamma^{2}\theta_{2}^{\prime }\right]+-2a^{2}\gamma^{2}\theta_{2}=0\text{.}$$

Where $C_{1}={\left(1-\varphi \right)}^{2.5},C_{2}=\left(1-\varphi \right)+\varphi \frac{\rho_{{Fe}_{3}O_{4}}}{\rho_{blood}}$ , $C_{3}=\frac{K_{blood}}{K_{{Fe}_{3}O_{4}}}\left[\left(1-\varphi \right)+\varphi \frac{{\left(\rho C_{p}\right)}_{{Fe}_{3}O_{4}}}{{\left(\rho C_{p}\right)}_{blood}}\right]$ and $\frac{K_{blood}}{K_{{Fe}_{3}O_{4}}}=\frac{\left(K_{{Fe}_{3}O_{4}}+2K_{blood}\right)-2\varphi \left(K_{blood}-K_{{Fe}_{3}O_{4}}\right)}{\left(K_{{Fe}_{3}O_{4}}+2K_{blood}\right)+\varphi \left(K_{blood}-K_{{Fe}_{3}O_{4}}\right)}$, also Pr is the Prandtl number and γ is a curvature parameter. The thermophysical characteristics for blood base fluid and Iron oxide nanoparticle is provided in Table 1 [34,36].

**Table 1:** The physical property of modeled components.

Property	Symbol	Blood	Irod Oxide
x	ρ	1063	5200
Dynamic viscosity	μ	0.003	–
Thermal conductivity	K	0.52	6
Heat Capacity	$C_{p}$	3746	670

Boundary conditions

The B.C.‘s includes wall, outlet, inlet and wall thermal insulation.

Inlet:

The rate of blood supply, or velocity is simulated on the inlet of AA. The volume of blood at the inlet can be control by velocity magnitude and inflow cross section area. The inlet boundary condition is $u={-U}_{0}n$. Here $U_{0}$ is the normal inflowing velocity having value 0.8 ms^−1^.

Outlet:

At the outlet, the pressure of blood inside the artery is taken in addition to more realistic manner to the simulation. In Fig. 2, it can be seen that there are two outlets opposite to the inlet from where the blood is flowing outside. The equation on the outlet in COMSOL is $\left[-PI+K\right]n=-\hat{P_{0}}n$, where $\hat{P_{0}}\leq P_{0}$. Here $p_{0}=12000pa$ is suppress backflow.

Wall:

The blood can't flow along the wall Since it sticks with wall because of the viscosity. So that, the boundary conditions along the wall are u=0,w=0, and there is no slip on the wall. The obtained thermal insulation on the boundary from COMSOL is −n.q=0.

## 3 Numerical procedure and code validation

The numerical computation which is based on finite element discretization is applied to obtain the solution. This discretization contains different functions for different shapes. A numerical approach was used to solve the energy equation and momentum equation because they can't be resolved analytically because of the nonlinear nature of the equations. The finite element method is applied to find a more precise approximation. The finite element method is one of the most remarkable numerical methods and a widely used approach to handle boundary value problems. Utilizing the Newton's method, the basic discrete nonlinear set of equations was linearized. The linearized inner systems were resolved by PARDISO, a direct solver that makes utilizes LU matrix factorization to reduce the number of iterations necessary to reach the desired level of convergence. Therefore, fewer iterations are necessary [37,38,39,40,41,42,43,44,45]. In interconnect architectures, this technique can be used to simulate and describe a variety of physical operations. The following are the six crucial phases in the finite element technique:

i) Discretization of the domain;
ii) Develop local equations for finite elements;
iii) Put together regional contributions to create a global matrix;
iv) Incorporate the initial and boundary conditions;
v) Fix the created system;
vi) Post-processing.

**Fig. 3:**
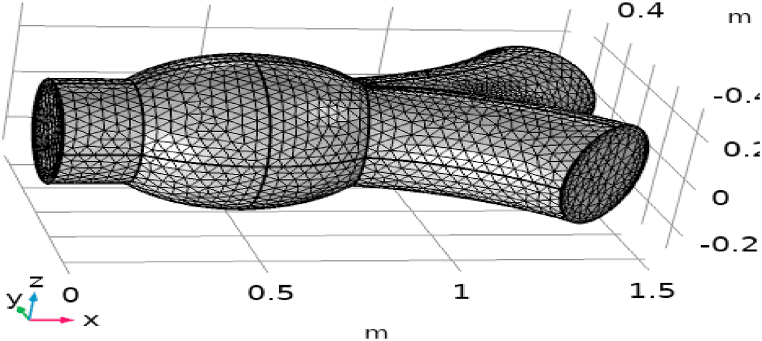
Mesh illustration of geometry.

Mesh has the great importance in the computational type of fluid dynamics. The convergence and accuracy of the solution is depending on the type of mesh. The normal physics-controlled mesh function is generated automatically in COMSOL as shown in Fig. 3.

## 4 Results and outcomes

The computational research of flow of blood inside the abdominal aorta is an important and difficult Concern. The most common illness in the arterial system is AAA, which is caused by the infection in the aorta and toughening of blood vessel walls. A simulation was performed to assess the pressure, temperature, and velocity of blood flowing across abdominal aorta in the appearance of AAA when nanoparticles are added. The inclusion of nanoparticles changed the material characteristics of blood, like as dynamic viscosity, density, heating value, and conductivity, causing the simulation outcomes to change. Nusselt number alteration was used to study the heat transfer phenomenon.

**Fig. 4:**
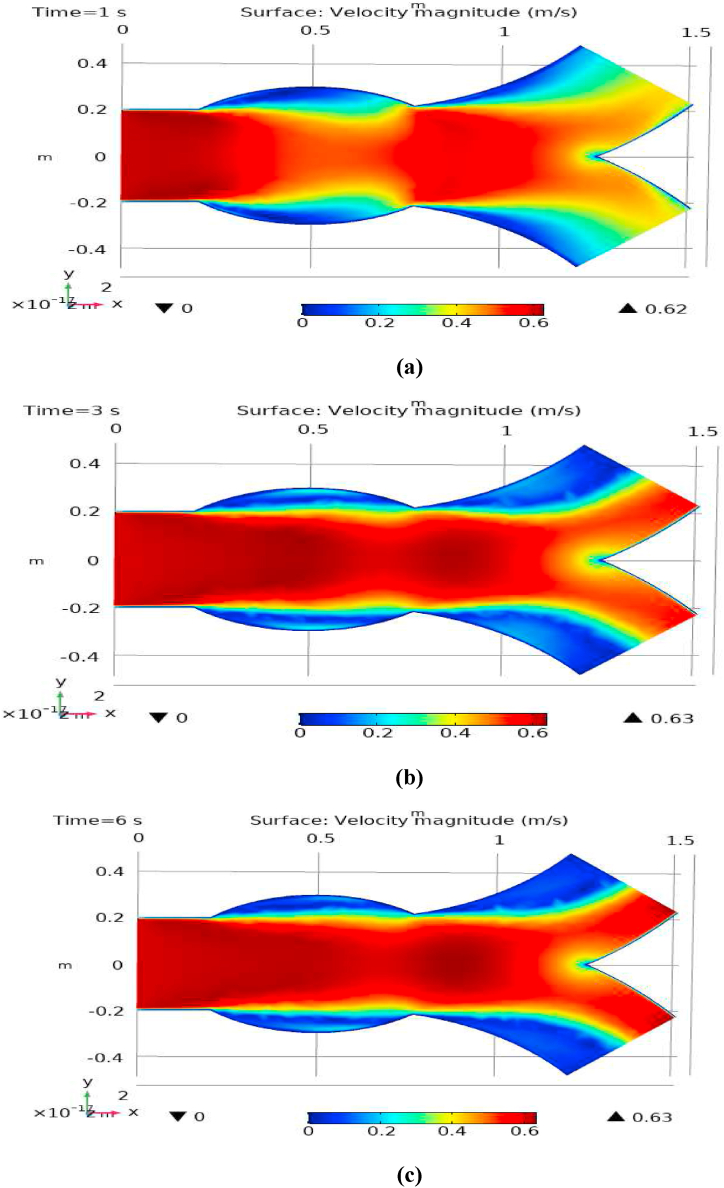
(a)–(c). Velocity plane graph for flow of blood nano-fluid inside AAA at different values of time.

**Fig. 5:**
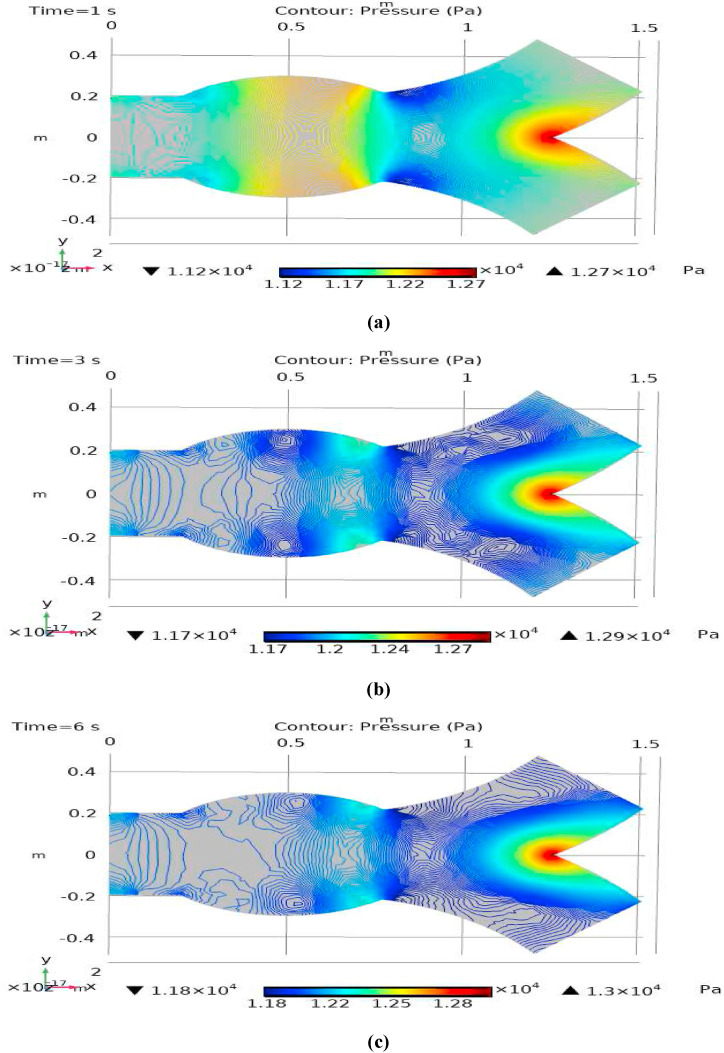
(a)–(c). Pressure plane graph for flow of blood nano-fluid inside AAA at different values of time.

**Fig. 6:**
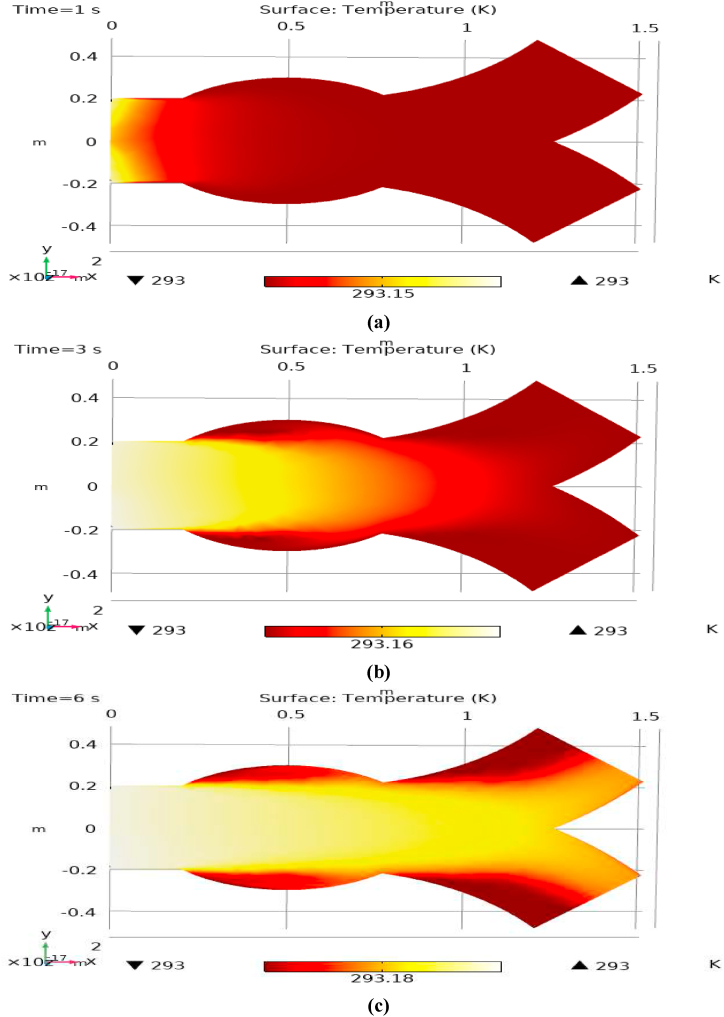
(a)–(c). Temperature plane graph for flow of blood nano-fluid inside AAA at different values of time.

Fig. 1 represents the actual condition of normal and aneurysmal abdominal aorta. Fig. 2 shows the constructed geometrical model for AAA. In which the one inlet, two outlet and aneurysm are mentioned. Fig. 3 is the mesh configuration, which shows the normal and finite size elements of constructed model. The wall of AAA is thermally insulated. Fig. 4, Fig. 5, Fig. 6 describe the velocity, pressure and temperature variations at different values of time like t=1s,t=3s and t=6s. Fig. 4(a–c) display the velocity planes inside the abdominal aortic aneurysm. It can be seen that the velocity is increasing with time. Fig. 5(a–c) explain the pressure inside the abdominal aortic aneurysm. The maximum pressure value is increasing with time. It depicts that the pressure is different at different points inside the abdominal aortic aneurysm. The pressure is normal at the start of the aneurysm and increasing up to the mid of the aneurysm and decreasing after that. Fig. 6(a–c) show the temperature planes for different values of t. The maximum value of temperature is same throughout the flow but it changes inside the aorta for different value of time.

**Fig. 7:**
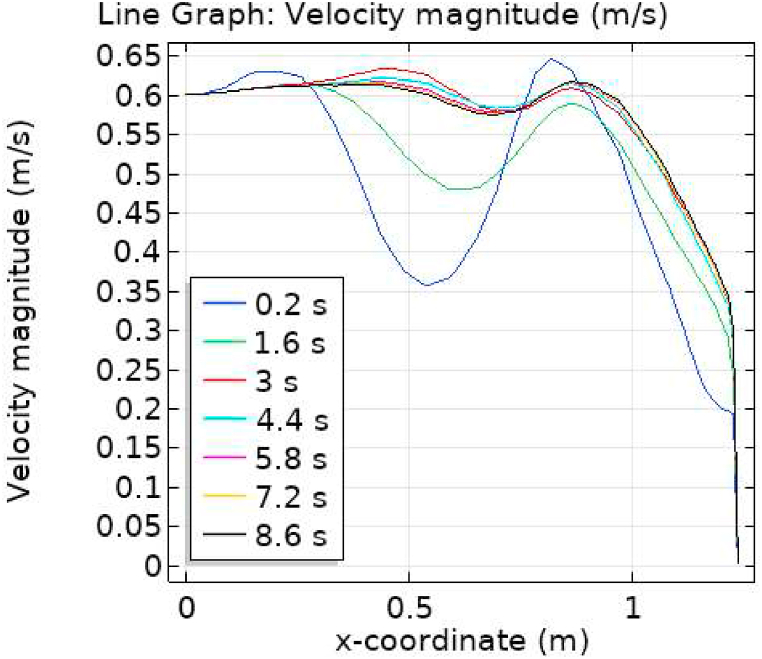
Velocity profile for flow of blood without nano-particles inside AAA at different values of time.

**Fig. 8:**
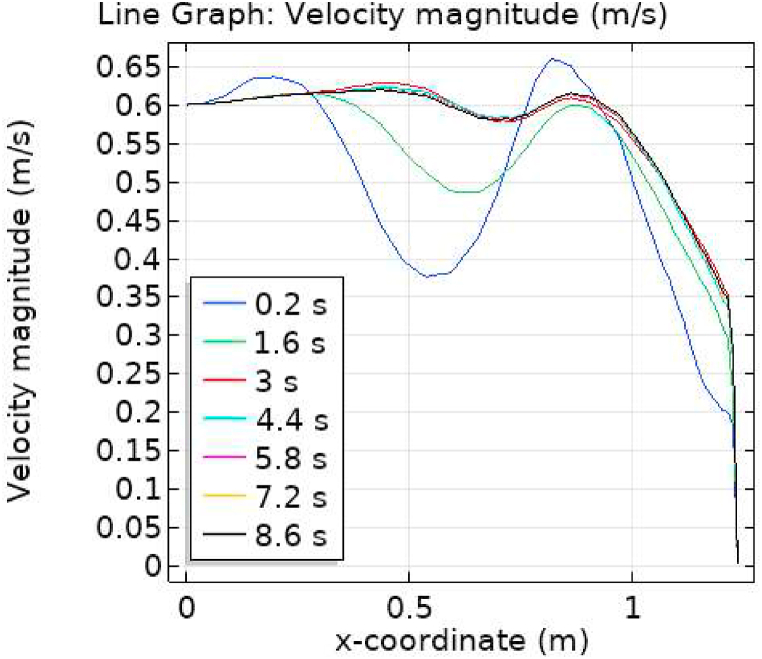
Velocity profile for flow of blood nano-fluid inside AAA at different values of time.

**Fig. 9:**
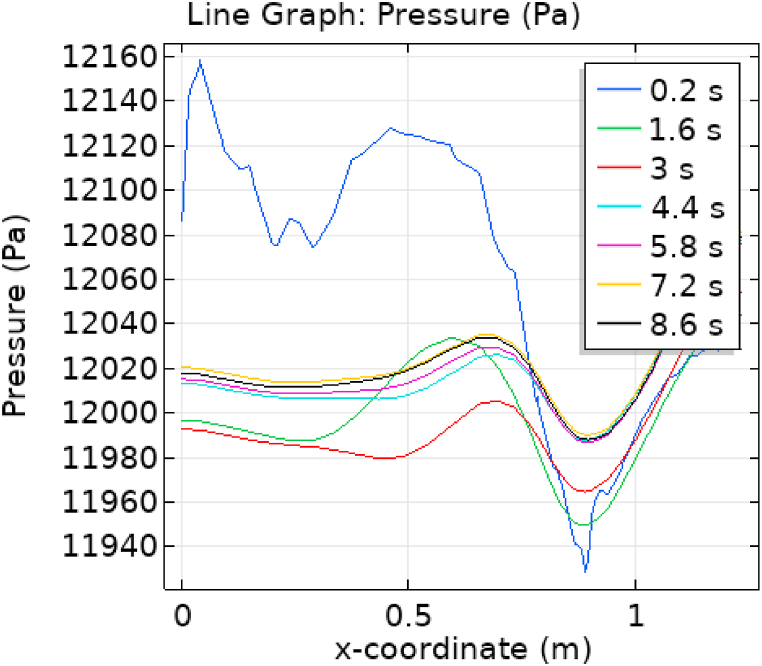
Pressure profile for flow of blood without nano-particles inside AAA at different values of time.

**Fig. 10:**
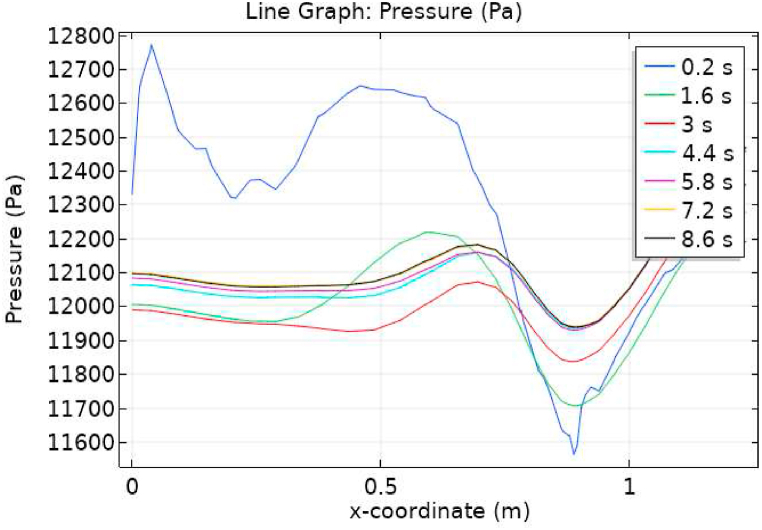
Pressure profile for flow of blood nano-fluid inside AAA at different values of time.

**Fig. 11:**
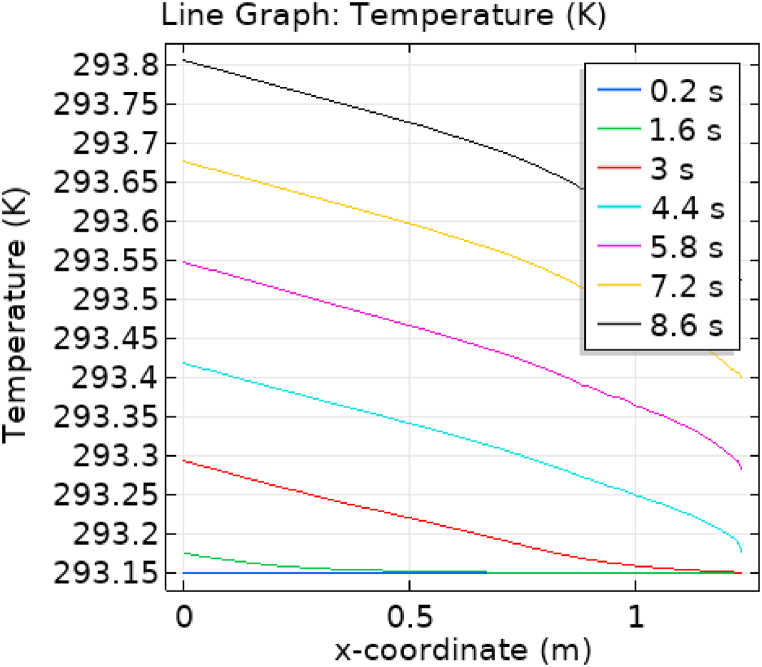
Temperature profile for flow of blood without nano-particles inside AAA at different values of time.

**Fig. 12:**
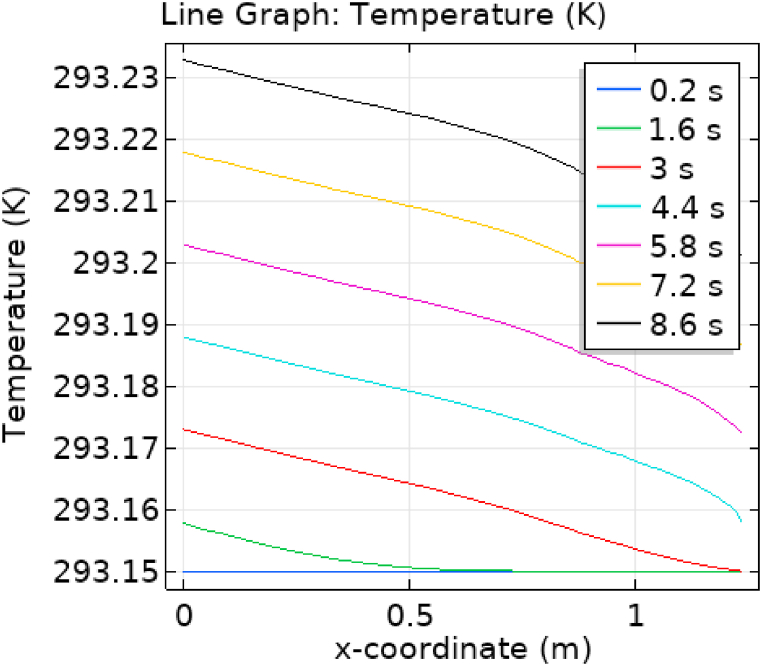
Temperature profile for flow of blood nano-fluid inside AAA at different values of time.

**Fig. 13:**
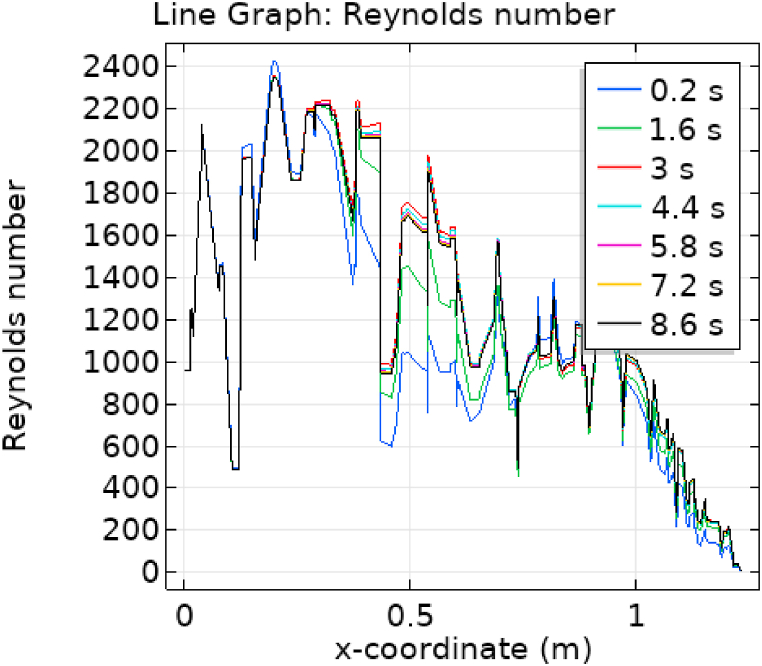
Reynold number for flow of blood without nano-particles inside AAA at different values of time.

**Fig. 14:**
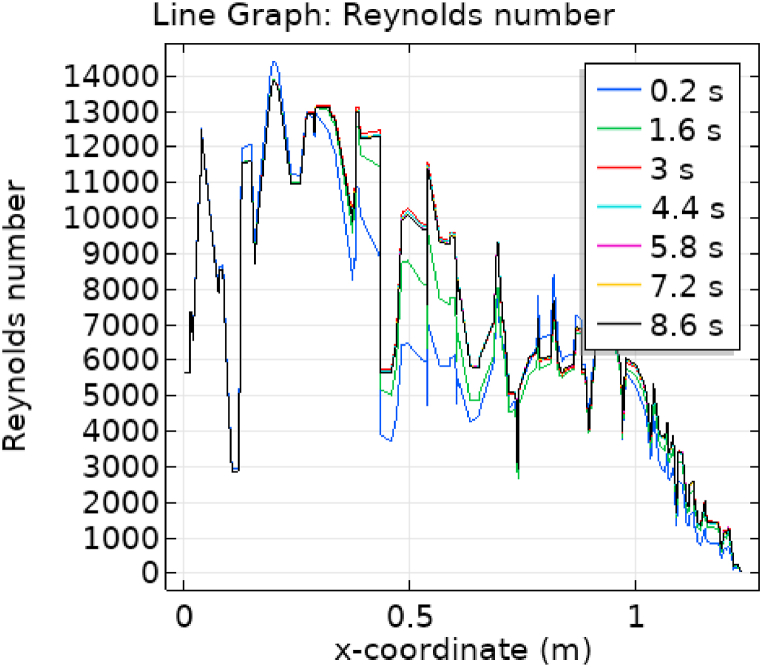
Reynold number for flow of blood nano-fluid inside AAA at different values of time.

Fig. 7, Fig. 8 represent the velocity profiles without and within the nanoparticles. It can be seen that the velocity is increasing along x-axis till the mid of aneurysmal region then suddenly it is decreasing. After decreasing it is again increasing at the end of the aneurysmal region. The velocity is increasing with time. But when we added nanoparticles in the blood the velocity shows the same behavior along the x-axis but it is controlled with respect to time. Fig. 9, Fig. 10 depict the pressure profiles excluding and including nanoparticles. The pressure is decreasing from start of the aorta to the mid of the aneurysm, then after increasing from mid to end of the aneurysm it is again decreasing. It is increasing with time. When the nanoparticles added the overall pressure increased. The pressure value was below 12000 without adding nanoparticles and it becomes 12000 when we added nanoparticles. Fig. 11, Fig. 12 show the temperature line graphs without and within nanoparticles. The temperature is decreasing along the x-axis and increasing with time. The maximum value of temperature decreased when the nanoparticles have been added. Fig. 13, Fig. 14 represent the Reynold number profile. The Reynold number decreasing along the x-axis. The value of Reynold number at the start is 1000 and when the nanoparticles are added the Reynold number value is increased up to 5500. The maximum value of Reynold number without and within nanoparticle is 2400 and 14000 respectively.

## 5 Conclusion

We glanced into the impacts of utilized nanoparticles in flow of blood through such an infected abdominal aorta. This study's primary purpose was to explain the Simulation result for pressure, temperature, and velocity into the abdominal aorta's narrow section. According to the outcomes, the addition of Iron Oxide nanoparticles prevented overheating and disfavored the highest velocity. Our modelling may be used to acquire a more accurate view of hemodynamics. Some of the most important findings are:

• The study of laminar flow described the velocity of the flow of blood is controlled by adding the nanoparticles. The maximum value of velocity is increasing with time.
• The aneurysm decreased the pressure but it is increasing and stable when we add iron oxide nanoparticles. It is increasing with time.
• The temperature is decreased by adding the iron oxid nanoparticles. It is increasing with time and decreasing along the x-axis.
• We can investigate the physical properties, including the skin friction, as well as solve problem by utilizing magnetohydrodynamic and radiation impacts to determine the causes of aneurysm, which may aid in the diagnosis of AAA.
• When iron oxide nano particles penetrate into a patient's bloodstream, they can change the characteristics of the blood or the walls of blood vessels thereby affecting the flow of blood. For instance, iron oxide nano particles enhance the blood viscosity, which can change the velocity of flow of blood and shear stress.

## Author contribution statement

Azad Hussain: Conceived and designed the analysis.

Muhammad Naveel Riaz Dar: Analyzed and interpreted the data; Wrote the paper.

Nashmi H. Alrasheed, Khalil Hajlaoui, Mohamed Bechir Ben Hamida: Contributed analysis tools or data.

## Data availability statement

Data will be made available on request.

## Declaration of competing interest

The authors declare that they have no known competing financial interests or personal relationships that could have appeared to influence the work reported in this paper.
